# Multiple Thrombectomies in the Same Patient within One Month: Case Report of a Patient with Trousseau Syndrome and Acute Ischemic Stroke

**DOI:** 10.3390/brainsci10090590

**Published:** 2020-08-26

**Authors:** Kurt Cicilioni, Brian Cristiano, J. Paul Jacobson, Daniel Hoss, Matthew Lund, Shauna Cheung, Justin Dye

**Affiliations:** 1 Department of Radiology, Loma Linda University Medical Center, Loma Linda, CA 92354, USA; bcristiano@llu.edu (B.C.); PJacobson@llu.edu (J.P.J.); DHoss@llu.edu (D.H.); 2 Division of Interventional Neuroradiology, Loma Linda University Medical Center, Loma Linda, CA 92354, USA; JDye@llu.edu; 3 Loma Linda University School of Medicine, Loma Linda, CA 92354, USA; mlund@llu.edu; 4 Department of Neurology, Loma Linda University Medical Center, Loma Linda, CA 92354, USA; SHCheung@llu.edu; 5 Department of Neurological Surgery, Loma Linda University Medical Center, Loma Linda, CA 92354, USA

**Keywords:** acute ischemic stroke, Trousseau syndrome, thrombectomy

## Abstract

Background and Importance: Since Trousseau’s initial publication, the development of thromboembolic events related to malignancy has been well established. The pathophysiology of this is understood to be through activation of the coagulation cascade through neoplastic cells themselves or the therapy initiated (chemotherapy or surgery). To date, there have been a variety of studies, such as the OASIS-CANCER trial, which highlight the relationship of hypercoagulability to ischemic stroke. Despite these efforts, clear evidence is lacking for the utilization of antiplatelet or anticoagulation therapy in the secondary prevention of stroke following mechanical thrombectomy in patients with suspected or confirmed malignancy. Clinical Presentation: A 71-year-old female with a history of immune thrombocytopenia, diabetes mellitus, and hypertension who was undergoing an evaluation for a lung nodule, later determined to be adenocarcinoma of the lung, underwent three successful mechanical thrombectomies for acute ischemic stroke with large vessel occlusion over a one month period. This patient had improved National Institutes of Health Stroke Scale (NIHSS) scores following each of her thrombectomies. However, her history of immune thrombocytopenia and underlying malignancy complicated her discharge medication regimen following each of her thrombectomies and may have contributed to her repeat strokes. Conclusion: Clear guidance is lacking regarding the utilization of antiplatelet and anticoagulation therapy in patients with suspected or confirmed malignancy following mechanical thrombectomy. Review of the literature suggests that controlling a patient’s hypercoagulability may lead to improved clinical outcomes, but further clinical trials are warranted.

## 1. Introduction

Since Trousseau’s initial publication, the development of thromboembolic events related to malignancy has been well established. The pathophysiology of this hypercoagulable state is thought to be through activation of the coagulation cascade by neoplastic cells themselves or secondary to the therapy initiated (chemotherapy and/or surgery) [1,2].

To date, there have been a variety of studies which highlight the relationship of hypercoagulability and ischemic stroke [3,4]. Unfortunately, the majority of these studies have significant limitations of power and generalizability in the secondary prevention of stroke. However, the need for recurrent mechanical thrombectomy within a narrow time frame in a patient with Trousseau’s syndrome has yet to be reported.

The current report describes a patient who was undergoing work up for mucin adenocarcinoma of the lung at the time of ischemic stroke presentation. The patient went on to require three mechanical thrombectomies over the course of one month. This case highlights the importance of secondary stroke prevention in patients with Trousseau syndrome.

## 2. The Case

A 71-year-old female with a past medical history significant for a 10 plus year history of benign idiopathic thrombocytopenia, controlled type II diabetes mellitus, hypertension and no prior stroke history had a lung nodule incidentally detected at an outside hospital in September 2019. At the time of initial presentation to our institution, the lung nodule had not yet been biopsied.

**Figure 1:**
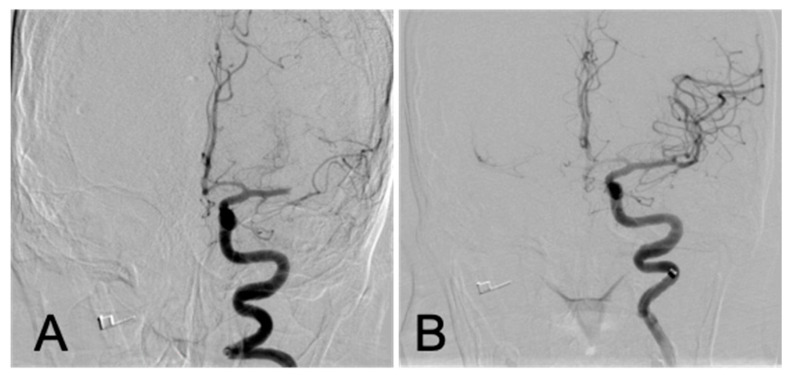
First thrombectomy, PA views: left M1 middle cerebral artery occlusion before (**A**) and after (**B**) successful mechanical thrombectomy.

During the second week of November 2019, the patient presented with acute onset dizziness, facial asymmetry, and aphasia with a National Institutes of Health Stroke Scale (NIHSS) score of 12. She was a candidate for, and received, intravenous tPA (tissue-type plasminogen activator) and computerized tomography (CT) angiography of the head revealed a left M1 segment middle cerebral artery (MCA) occlusion. The patient underwent mechanical thrombectomy, with single pass aspiration, with near complete restoration of MCA territory antegrade flow - achieving a TICI (Thrombolysis in Cerebral Infarction) score of 2C (Figure 1). Her NIHSS improved to five at 24 hours post thrombectomy and she was subsequently transferred to an outside hospital two days later for ongoing stroke care. At the time of transfer, the decision was made to initiate aspirin 81 mg and hold therapeutic doses of low-molecular weight heparin despite her history of ITP (immune thrombocytopenia). The patient was discharged from the outside hospital seven days post thrombectomy. During the patient’s hospitalization a complete stroke work-up was performed. An echocardiogram demonstrated a good ejection fraction, no patent foramen ovale, no valvular abnormality and no thrombi. CT angiography of the neck showed no evidence of carotid bifurcation disease, and thus a specific stroke etiology was not identified. At the time of discharge from the outside hospital, the patient was continued on aspirin 81 mg following recommendations from hematology/oncology.

**Figure 2:**
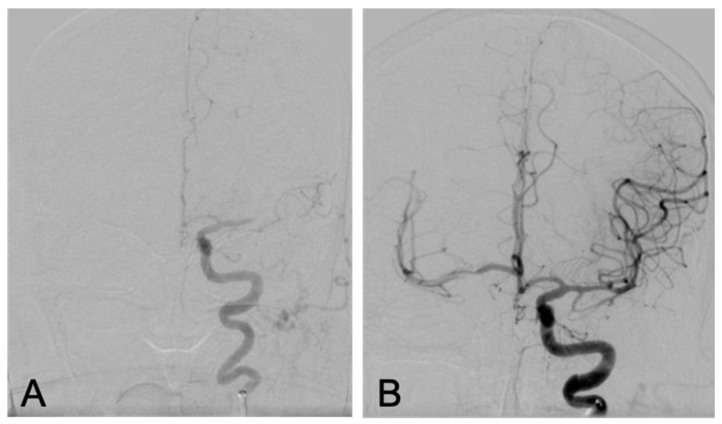
Second Thrombectomy, PA views: left M1 middle cerebral artery occlusion before (**A**) and after (**B**) successful mechanical thrombectomy.

**Figure 3:**
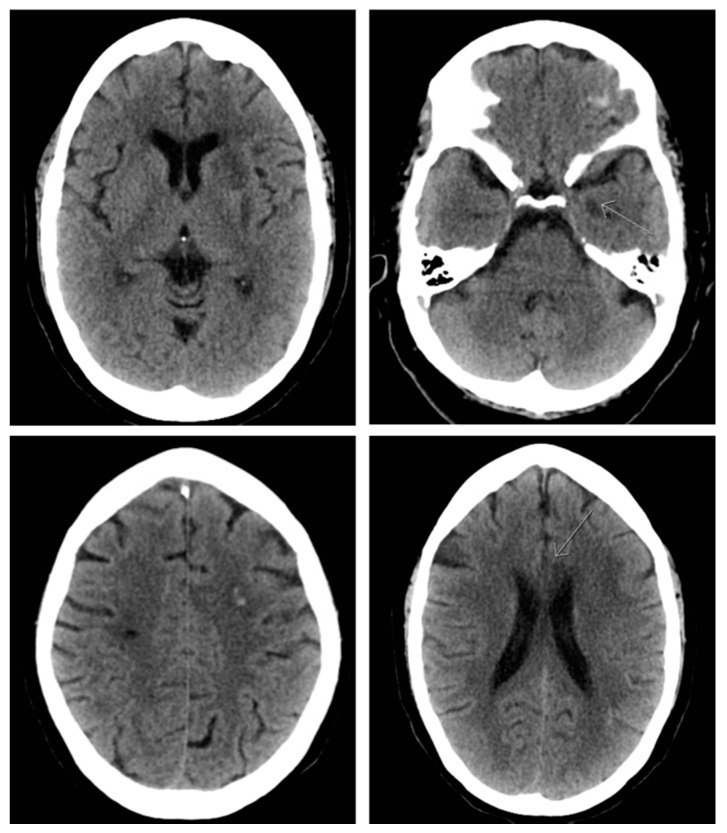
Non-contrast head CT showing multiple areas of small petechial hemorrhage 24 hours following the second mechanical thrombectomy.

Nearly two weeks later the patient again presented with recurrent symptoms including right-sided facial droop and right-sided weakness. NIHSS was 11. At this time, tPA was not administered given her recent stroke being an absolute contraindication. CT angiography revealed a recurrent left M1 segment MCA occlusion. A successful mechanical embolectomy was performed with near complete restoration of antegrade flow in the MCA territory after a direct aspiration thrombectomy achieving a TICI score of 2C (Figure 2). The 24-h post procedure head CT, the patient was noted to develop petechial hemorrhages (left basal ganglia/internal capsule, paramedian left frontal lobe, body of corpus callosum and mesial left temporal lobe) (Figure 3). Aspirin was discontinued due to hemorrhage and a platelet count of 42,000. Once clinically stable, the patient was again transferred to an outside hospital with an NIHSS of three.

At the outside hospital, the patient was started on aspirin 325 mg following discussion with the hematology/oncology team and was discharged to home. Two days following discharge, the patient was evaluated by outpatient neurology who recommended the patient initiate low-molecular-weight heparin following clearance by hematology/oncology. However, the patient refused low-molecular weight heparin and remained on aspirin 325 mg only.

**Figure 4:**
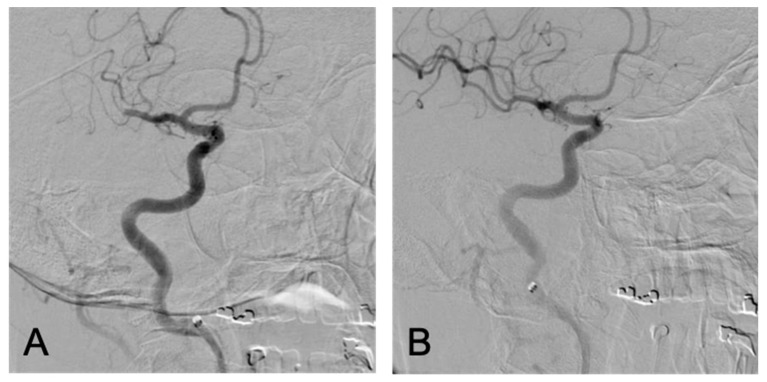
Third Thrombectomy, PA views: right M2 dominant inferior division occlusion before (**A**) and after (**B**) successful thrombectomy.

Six days following the of the patient’s second thrombectomy, the patient again returned to our institution with symptoms of aphasia and left-sided facial droop. NIHSS was determined to be eight. A new right M2 dominant inferior division occlusion was identified and a third successful thrombectomy was performed using a combination of retrievable stent and aspiration with a TICI score of 2C. After the thrombectomy, her NIHSS improved to two (Figure 4). Follow-up head CT at 24 h was negative for hemorrhagic transformation. Following her third ischemic stroke, aspirin and low-molecular-weight heparin were both initiated two days post thrombectomy. She has since remained on aspirin, while low-molecular-weight heparin was discontinued at the time of discharge.

The patient’s suspicious lung nodule was subsequently biopsied in December 2019 and confirmed to be EGFR+ mucinous adenocarcinoma. In February 2020, the patient was noted to be improving clinically and was initiated on osimertinib (Tagrisso) for treatment of her cancer.

## 3. Discussion

The mechanisms driving the increased risk of embolic and ischemic stroke in cancer patients is multifactorial and has been linked to the extent of the disease, tumor biology, local and systemic inflammation, cancer therapeutics, and patient-related factors [1,4]. The risk of stroke in cancer patients also seems to be associated with the aggressiveness of the cancer; with lung, pancreatic, colorectal, and primary brain carrying the highest risk [5].

Basic science research of late has been focused on the role that inflammation plays in the pathophysiologic mechanisms of cancer associated stroke. Specifically, researchers have recently proposed the role of extracellular vesicles (EVs) and neutrophil extracellular traps (NETs), which are networks of extracellular fibers composed predominantly of DNA from neutrophils. The current understanding is that cancer cells produce circulating EVs, tissue factor and cancer procoagulants along with other inflammatory cytokines. The increased circulating EVs subsequently lead to increased neutrophil activation and subsequent release of de-condensed chromatin and formation of NETs. The NETs are proposed to serve as a scaffolding for the collection of red blood cells, platelets, fibrinogen, and platelet adhesion molecules along with activating both intrinsic and extrinsic coagulation pathways leading to downstream thrombosis. This has led to further research to evaluate NET specific biomarkers and their usefulness in determining patients’ risk for pro-coagulable state [6,7,8,9].

More recently, the effects of multiple mechanical thrombectomies in patients with recurrent large vessel occlusions has been evaluated. Pirson, et al. using the registry from the MR CLEAN trial, retrospectively analyzed patients with anterior circulation recurrent acute ischemic stroke, with a focus on timing between initial and repeat thrombectomy and clinical outcomes based on modified Rankin Scale (mRS) at 90 days. The study included 3928 patients over a 15 year period. They found that only 27 patients required repeat mechanical thrombectomy. The majority of these cases had an underlying cardioembolic source for their stroke (*n* = 18, 67%). At 90 days, 44% of patients had achieved functional independence. The authors concluded that recurrent mechanical thrombectomy appears to be safe [10]. However, despite the large sample size, the number of patients with repeat thrombectomy was very small. Due to the rarity of repeat thrombectomy, further study of this entity is necessary. Further, the utilization of repeat mechanical thrombectomy in the setting of malignancy remains to be clearly defined.

Guidelines on the utilization of antithrombotic therapy in the setting of acute ischemic stroke intervention in patients with underlying or suspected malignancy have not been established. Regarding secondary prevention in this patient population, common practice is to initiate low molecular weight heparin [11,12,13,14]. This common practice is based on studies designed for the management of recurrent deep venous thromboembolism as opposed to acute ischemic stroke. Recently, evidence from the Hokusai VTE Cancer trial and Select-D Pilot trial showed noninferiority of oral factor Xa inhibitors when compared to LMWHs with respect to the outcome of recurrent venous thromboembolism or major bleeding [11,15,16]. In addition, efforts including multiple meta-analyses have been performed to establish guidelines for the role of anti-platelet therapy in primary, secondary, and tertiary prevention of stroke [5]. However, these have largely been unsuccessful in identifying clear evidence, resulting in significant variations in practice among clinicians, which is further compounded in the setting of malignancy. Among others, the decision of whether or not to initiate LMWH is made through clinical judgement by weighing the risks, benefits, type of cancer, presence of brain metastasis, blood count, current cancer treatments, and renal impairment.

At this time, research has been focused on evaluating the effectiveness of oral anticoagulants as well as low molecular weight heparin among cancer patients for secondary stroke prevention [17,18,19,20,21]. Among clinicians and researchers, concern has been raised regarding oral anticoagulants and their possible interaction with chemotherapeutic agents used for supportive care via the CYP3A4 enzyme and/or P-glycoprotein transporter, as well as the possibility of impaired absorption in vomiting patients or chemotherapy-induced intestinal mucosal defects [16]. The alternative antithrombotic option for secondary prevention of stroke is antiplatelet therapy, typically aspirin. There are concerns that antiplatelet therapy may not sufficiently offset cancer-mediated hypercoagulability which is a major factor in cancer-associated strokes [12]. Currently, minimal research exists evaluating anticoagulation compared to antiplatelet therapy in patients with cancer and acute ischemic stroke.

Trials such as the TEACH and OASIS-CANCER trials have brought to light the need for further investigation into optimal antithrombotic therapy for ischemic stroke in patients with underlying cancer [6,22]. In the TEACH trial, outcomes of patients who received therapeutic doses of low molecular weight heparin compared to aspirin were noted to have no significant difference in clinical outcomes specifically relating to rates of major bleeding, ischemic stroke, or overall survival. The power of the study was limited by a small sample size. However, the authors did propose that many of the ischemic strokes experienced by patients with cancer are associated with cancer-mediated hypercoagulability and therefore anticoagulants such as low molecular weight heparinhave the potential to be more effective in patients with Trousseau’s syndrome.

In the OASIS-CANCER trial, patients either received warfarin, direct oral anticoagulants, or low molecular weight heparin. Patients with active malignancy were assessed for the recurrence of stroke or systemic embolism. The study included 268 patients and determined that successful correction of hypercoagulability, based on D-dimer values, was associated with improved survival rates [6].

In the setting of mechanical thrombectomy for acute ischemic stroke, the degree of symptomatic intracranial hemorrhage plays a significant role in patient prognosis [23]. Current guidelines recommend that once hemorrhagic conversion occurs, all antiplatelet/anticoagulation therapy be held along with strict blood pressure control. Among most clinicians, based on the stability of the patient, aspirin can be initiated within the first two days following hemorrhage, while anticoagulation is held for a minimum of two weeks before re-initiation is considered [24]. However, no clear guidance has been established on the use of antiplatelet/anticoagulation in the setting of hemorrhagic transformation post thrombectomy in patients with an underlying malignancy, as in our case.

## 4. Conclusions

Despite initial efforts, clear guidance is lacking regarding the utilization of antithrombotic therapy in patients with suspected or confirmed malignancy following mechanical thrombectomy. Review of the literature suggests that controlling a patient’s hypercoagulability with antithrombotic therapy can lead to improved clinical outcomes. However, further research is warranted to establish the best antithrombotic medication and time course following acute stroke intervention.
